# Mutation of p53 Acetylation Protects Against Angiotensin-II-Induced Cardiac Dysfunction and Fibrosis

**DOI:** 10.3390/ijms26199668

**Published:** 2025-10-03

**Authors:** Aubrey C. Cantrell, Quinesha A. Williams, Jian-Xiong Chen, Heng Zeng

**Affiliations:** Department of Pharmacology and Toxicology, University of Mississippi Medical Center, 2500 North State Street, Jackson, MS 39216, USA; aubreycantrell7@gmail.com (A.C.C.); qawilliams@umc.edu (Q.A.W.); jchen3@umc.edu (J.-X.C.)

**Keywords:** p53 acetylation, heart failure, hypertension, cardiac fibrosis

## Abstract

Hypertension is a major risk factor for heart failure. Acetylation of p53 is known to regulate its activities. We have previously identified that p53 acetylation is required for cardiac remodeling in a mouse model of pressure overload-induced heart failure. Acetylation mutant p53 (p53aceKO) mice have been shown to have the ability to regulate SIRT3 KO-induced cardiac fibrosis. In the present study, we hypothesized that p53aceKO mice would exhibit cardiac protection and blunt cardiac fibrosis when subjected to Ang-II-induced hypertension. Control and p53aceKO mice received either a micro-osmotic pump implant administering Ang-II for 28 days or a sham procedure. Blood pressure was measured weekly, and echocardiography was performed every two weeks. Mice were euthanized and hearts were processed for histological analysis. While both control and p53aceKO mice receiving Ang-II exhibit increased systolic and diastolic blood pressures, control mice also demonstrate increases in ejection fraction and fractional shortening compared to the sham, while p53aceKO mice do not. Furthermore, control mice receiving Ang-II exhibit decreased left ventricular diameter and volume at end-systole and end-diastole, as well as thickening of both the anterior and posterior walls, while p53aceKO mice exhibit no significant changes in any of these parameters. Additionally, p53aceKO mice do not exhibit the Ang-II infusion-induced cardiac fibrosis seen in control mice treated with Ang-II. Mutation of p53 acetylation is protective against Ang-II infusion-induced cardiac fibrosis and dysfunction in mice. Acetylated p53 may, therefore, be a novel therapeutic target to address complications in the heart associated with hypertension.

## 1. Introduction

Heart failure presents a major burden to society, affecting approximately 63 million people worldwide [1,2]. Hypertension is a significant risk factor for heart failure, and in the Framingham Heart Study, hypertension predated 91% of new-onset heart failure cases [3]. Therefore, identifying the underlying mechanisms of cardiac remodeling and dysfunction in response to hypertension could help prevent hypertension from progressing into heart failure.

Angiotensin-II (Ang-II) is a major part of the renin–angiotensin–aldosterone system that canonically regulates blood pressure [4]. Ang-II has been shown to induce cardiac fibrosis by activating cardiac fibroblasts via the Angiotensin-II type I receptor [5]. p53 has previously been shown to regulate the proliferation of cardiac fibroblasts in a model of pressure overload-induced cardiac remodeling [6]. Furthermore, p53 signaling has been implicated in the pathophysiology of diabetes-induced cardiac fibrosis [7]. p53 has previously been shown to be involved in Ang-II-induced cardiac hypertrophy [8]; however, to our knowledge, the therapeutic potential of blocking p53-mediated responses to Ang-II has yet to be investigated.

Acetylation mutant p53 (p53aceKO) mice have previously been extensively characterized in tumors [9]. In this model, the p53 protein loses some, but not all, of its normal functions; in particular, p53-mediated apoptosis, growth arrest, and ferroptosis are not activated in this model [9,10]. Deacetylation of p53 has been associated with reduced renal fibrosis caused by calcium oxalates via inhibition of the ferroptosis pathway [11]. Our lab has previously established that p53 acetylation is required for cardiac remodeling in a mouse model of heart failure [12]. At this point, the role of p53 acetylation in cardiac fibrosis is unclear. Therefore, we hypothesized that p53aceKO mice would be protected against cardiac fibrosis and dysfunction in response to Ang-II infusion.

## 2. Results

### 2.1. Both Control and p53aceKO Mice Exhibit Increased Blood Pressure Following Ang-II Infusion

**Figure 1:**
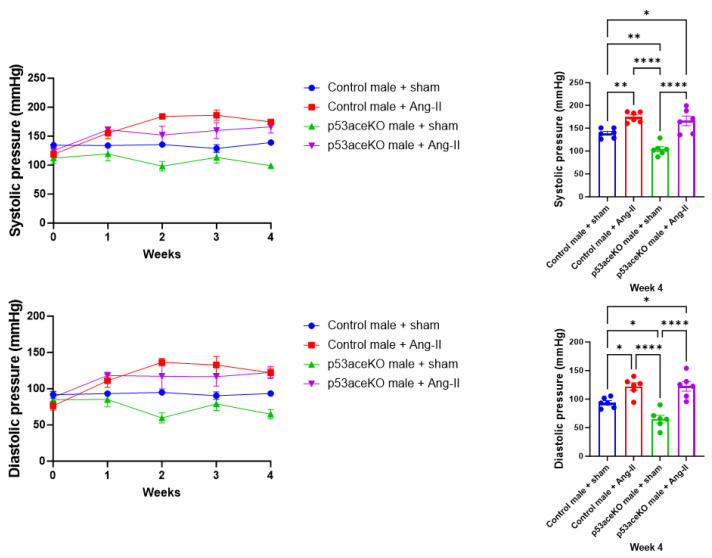
Both control and p53aceKO mice exhibit increased systolic and diastolic blood pressures in response to Ang-II. p53aceKO mice receiving sham treatment demonstrate slightly lower systolic and diastolic pressures compared to control mice. N = 6 mice per group, * *p* < 0.05, ** *p* < 0.01, **** *p* < 0.0001 using One-Way ANOVA with Tukey’s post hoc test.

Following four weeks of Ang-II infusion, both control and p53aceKO mice exhibit significant increases in systolic and diastolic blood pressures compared to their sham counterparts. Intriguingly, p53aceKO mice receiving sham procedures exhibit lower systolic and diastolic pressures compared to the controls (Figure 1).

### 2.2. p53aceKO Mice Have a Higher Heart Weight-to-Tibia Length Ratio at Baseline and Lose Less Weight over the Course of Ang-II Infusion than Control Mice

**Figure 2:**
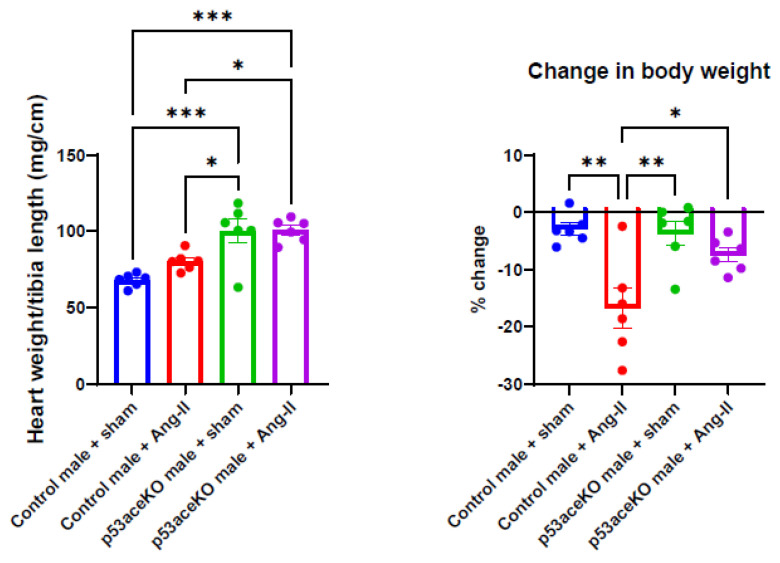
p53aceKO mice have a higher heart weight-to-tibia length ratio compared to control mice. Control mice demonstrate a trend towards an increase in heart weight to tibia length with Ang-II treatment, while p53aceKO mice do not. Control mice treated with Ang-II also lose weight compared to the sham, while p53aceKO mice do not. N = 6 mice per group, * *p* < 0.05, ** *p* < 0.01, *** *p* < 0.001 using One-Way ANOVA with Tukey’s post hoc test.

Interestingly, p53aceKO mice have a significantly higher heart weight-to-tibia length ratio compared to control mice. While control mice receiving Ang-II have a trend towards an increase in heart weight-to-tibia length ratio, p53aceKO mice receiving Ang-II do not. Heart weight-to-tibia length ratio is typically used to evaluate cardiac size relative to body size without using weight, as the tibia remains fairly consistent in length among strains of mice, while body weight can change significantly and is more dependent on experimental design (as seen by the degree to which mice lose weight in response to Ang-II infusion). Additionally, control mice receiving Ang-II have a significant loss in body weight compared to the sham, while p53aceKO mice receiving Ang-II do not (Figure 2).

### 2.3. p53aceKO Mice Do Not Exhibit the Ang-II Infusion-Induced Alterations in Cardiac Function Seen in Control Mice

**Figure 3:**
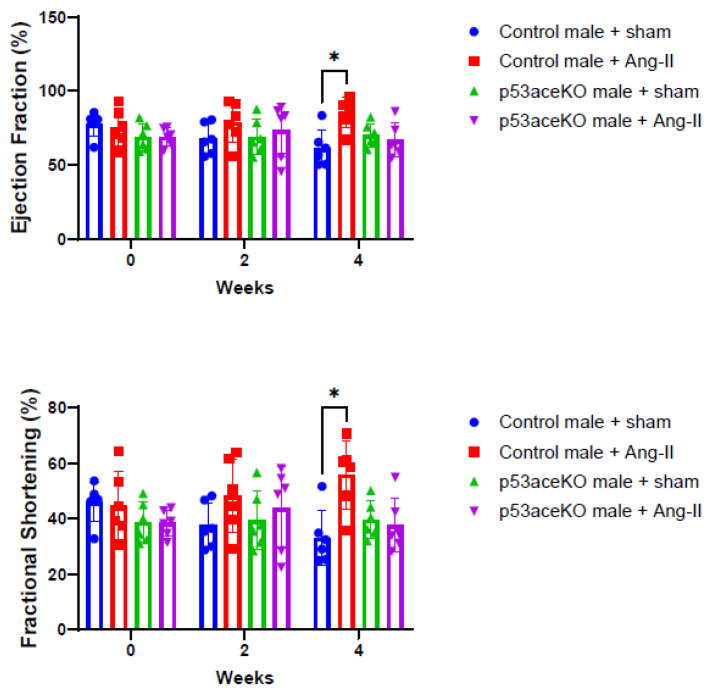
Control mice demonstrate increased EF and FS in response to Ang-II, while p53aceKO mice do not. N = 6 mice per group, * *p* < 0.05 using Two-Way ANOVA.

**Figure 4:**
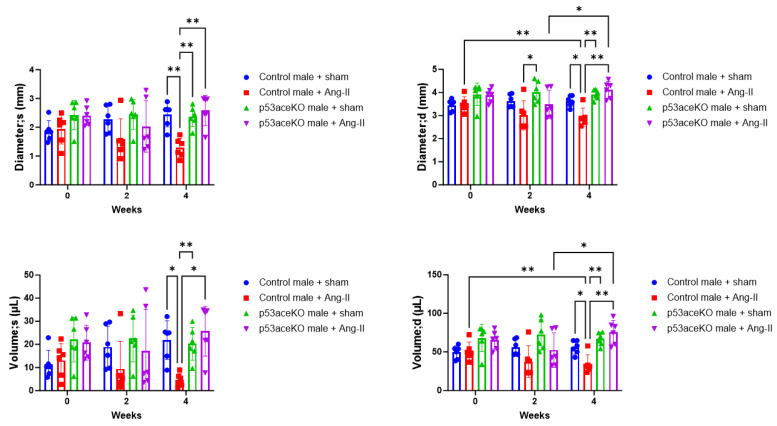
Control mice exhibit decreases in LV diameter and volume following Ang-II infusion, while p53aceKO mice do not. N = 6 mice per group, * *p* < 0.05, ** *p* < 0.01 using Two-Way ANOVA.

Control mice receiving Ang-II exhibit increased ejection fraction (EF) and fractional shortening (FS) compared to the sham, while p53aceKO mice do not (Figure 3). Furthermore, while control mice treated with Ang-II have significant decreases in left ventricular diameter and volume both at end-systole and end-diastole, p53aceKO mice receiving Ang-II demonstrate no significant alterations in any of these parameters (Figure 4). p53aceKO mice also exhibit no significant changes in left ventricular anterior wall thickness at end-systole (LVAW;s), in left ventricular anterior wall thickness at end-diastole (LVAW;d), in left ventricular posterior wall thickness at end-systole (LVPW;s), or left ventricular posterior wall thickness at end-diastole (LVPW;d) following Ang-II infusion, while control mice have significant increases in each of these following Ang-II infusion. Neither control nor p53aceKO mice receiving Ang-II demonstrated significant alterations in cardiac output (CO) or stroke volume (SV) (Table 1).

### 2.4. p53aceKO Mice Do Not Exhibit the Ang-II-Induced Cardiac Fibrosis Seen in Control Mice

**Figure 5:**
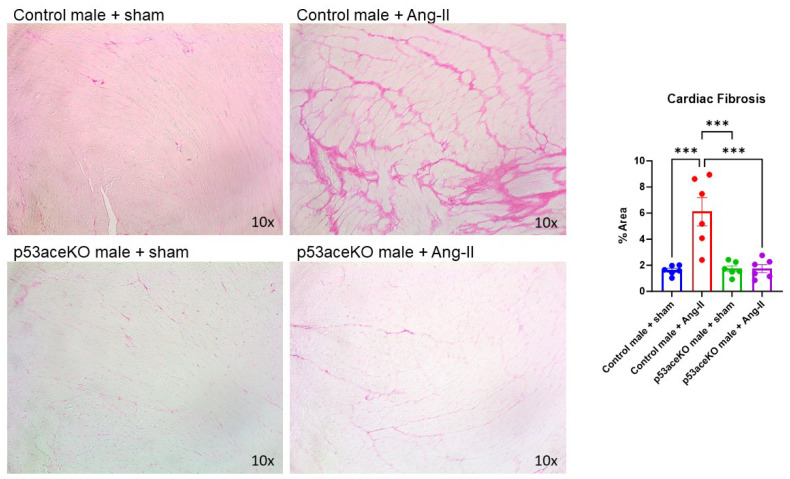
Histological analysis demonstrates that control mice receiving Ang-II have significant cardiac fibrosis compared to the sham, while p53aceKO mice receiving Ang-II do not. Magnification 10×, N = 6 mice per group, *** *p* < 0.001 using One-Way ANOVA with Tukey’s post hoc test.

Histological analysis demonstrated that, while control mice receiving Ang-II have significant cardiac fibrosis, p53aceKO mice receiving Ang-II do not (Figure 5).

## 3. Discussion

The present study identified for the first time that p53 acetylation mutation is protective against Ang-II infusion-induced cardiac dysfunction and fibrosis. The p53 acetylation mutation of p53aceKO mice has previously been extensively characterized [9]. This model exhibits disruptions in the apoptosis and growth arrest functions of p53, as well as its ability to regulate various proteins, and its ability to induce ferroptosis [9,10]. This is consistent with our previous study identifying that p53aceKO mice have preserved capillary density and coronary flow reserve following pressure overload [13].

Interestingly, p53aceKO mice receiving Ang-II did not lose as much weight compared to control mice treated with Ang-II. Control mice receiving Ang-II exhibited increases in EF and FS, due to hypertrophy of the LV walls leading to decreased LV diameter and volume both at end-systole and end-diastole. p53aceKO mice were seemingly protected from this effect, as they exhibited no changes in these parameters following Ang-II treatment despite their significant elevations in systolic and diastolic blood pressures. Histologically, p53aceKO mice did not exhibit increased fibrosis in the heart following Ang-II treatment, suggesting that mechanistically p53 acetylation mutation prevents cardiac remodeling. It stands to reason that this is due to the inhibition of p53 activity in this model, and more work is necessary to further elucidate whether one or more of the impaired pathways is responsible for cardiac fibrosis and dysfunction in response to Ang-II. Our lab has previously identified that p53aceKO in SIRT3 knockout mice reduced cardiac fibrosis and activation of the ferroptosis cell death pathway in this model [12]. We have also previously shown that p53aceKO mice exhibit improved endothelial cell function in a pressure overload-induced model of heart failure [13]. These prior studies support the idea that p53 acetylation may mediate cardiac protection against Ang-II-induced cardiac dysfunction and fibrosis via suppressing p53-mediated ferroptosis and improving vascular function in the heart. Further studies are necessary to sufficiently elucidate the precise mechanism by which p53 acetylation mediates these beneficial effects on the heart.

p53 has been shown to regulate the proliferation of cardiac fibroblasts in a model of pressure overload-induced heart failure, with p53 knockout resulting in hyperproliferation of cardiac fibroblasts and ultimately extensive cardiac fibrosis [6]. Because p53aceKO mice still express the p53 protein with many of its activities intact, it is possible that this model acts to suppress cardiac fibrosis in response to Ang-II infusion by suppressing cardiac fibroblast activity and proliferation. This is yet another pathway that remains to be elucidated, and the impacts of p53aceKO on specific cell types would benefit from in-depth study. P53 signaling in endothelial cells may also warrant further investigation, as this may be the basis for the lower systolic and diastolic blood pressures in p53aceKO mice receiving the sham procedure.

It is important to note that this study utilized only male mice, due to the known sex differences in response to Ang-II [14]. Future studies are necessary to determine whether the protective effects of p53 acetylation mutation are universal between sexes, or if this effect is exclusive to males. Furthermore, the p53aceKO mouse line is on the C57BL/6J background, whereas our control mice were of the 129S1/SvlmJ background. While both lines are considered viable wild-type models, it is possible that some of these differences could be exaggerated due to the difference in background, although the authors argue that the statistical significance of these results is unlikely to be substantially different in the event the experiments are repeated with 129S1/SvlmJ control mice.

A limitation of our study is that we did not investigate the underlying mechanisms by which p53aceKO attenuates Ang-II–induced cardiac fibrosis. In particular, we did not assess vascular leakage, endothelial proliferation, or inflammatory responses in the myocardium, all of which could provide mechanistic insights into the observed cardioprotective effects. Although our previous studies demonstrated that mutation of p53 acetylation preserves capillary density, improves coronary flow reserve, and reduces apoptosis, ferroptosis, and endothelial activation in pressure overload and SIRT3 deficiency-mediated cardiac fibrosis [12,13], whether similar mechanisms underlie the protective effects against Ang-II–induced fibrosis remains unknown. Future studies will be needed to elucidate these pathways and to determine whether vascular preservation and suppression of inflammation contribute to the antifibrotic actions of p53aceKO.

## 4. Methods

### 4.1. Experimental Animals

Mice expressing mutated p53 with arginine replacing lysine at amino acid residues K98, K117, K161, and K162 (p53^4KR^, p53aceKO) on the C57BL/6J background were provided by Dr Wei Gu at Columbia University. Wild-type control mice of the 129S1/SvImJ strain were obtained from The Jackson Laboratory (strain number 002448). Mice were all maintained in the Laboratory Animal Facility at the University of Mississippi Medical Center (UMMC), were fed laboratory standard chow and water, and were housed in individually ventilated cages. All protocols were approved by the Institutional Animal Care and Use Committee at UMMC (Protocol ID: 1564, 1189) and were in compliance with the National Institutes of Health Guide for the Care and Use of Laboratory Animals (NIH Pub. No. 85--23, Revised 1996). Because there are known sex differences in response to Ang-II infusion [14], this study utilized only male mice, with future studies to evaluate this pathway in females.

Power analysis was used to determine the number of mice required for statistically significant results. Mice received either a micro-osmotic pump implant or a sham procedure on Day 0. On Day 29, mice were weighed, euthanized and hearts excised, weighed, and fixed in 10% Neutral Buffer Formalin (Epredia #51401).

### 4.2. Micro-Osmotic Pump Implant or Sham Procedure

Male 5–8-month-old mice were randomly divided into groups as follows (N = 6 mice per group): control + sham, control + Ang-II, p53aceKO + sham, and p53aceKO + Ang-II. Ang-II was purchased from BACHEM (#4006473). Micro-osmotic pumps were purchased from Alzet (model 1004) and prepared according to the manufacturer’s instructions to deliver 1 μg/kg body weight/min of Ang-II for 28 days. Pump implant or sham procedures were performed on Day 0 as previously described [15]. Briefly, mice were anesthetized with isoflurane and the pump was implanted (midscapular region) subcutaneously. The sham procedure was identical except that no pump was implanted. Mice were administered carprofen at a dose of 5 mg/kg body weight subcutaneously after the procedure and for two days following the procedure.

### 4.3. Blood Pressure Measurement

Tail-cuff blood pressure measurement was performed without anesthesia using the CODA Non-Invasive Blood Pressure System (Kent Scientific). Mice were trained to become acclimated to restraint for 20–30 min per day for 4 days prior to initial measurement. Measurements were performed between 6:00 and 9:00 a.m., and 20 measurements were taken per mouse per day.

### 4.4. Transthoracic Echocardiography

Echocardiographic measurements were performed transthoracically on mice as previously described [16] on Days 0, 14, and 28. Mice were anesthetized via inhalation of 1–1.5% isoflurane mixed with 100% medical oxygen. Measurements were taken using the Vevo 3100 Preclinical Imaging Platform with an MX400 transducer (FUJIFILM Visual Sonics Inc., Toronto, ON, Canada). An acceptable heart rate of between 450 and 500 beats per minute was maintained for the duration of measurement. M-mode cine loop analysis was performed using Vevo LAB software 3.2.6 (FUJIFILM Visual Sonics Inc., Canada) to obtain ejection fraction (EF), fractional shortening (FS), and myocardial parameters, including left ventricle (LV) end-systolic diameter (Diameter;s); LV end-diastolic diameter (Diameter;d); LV end-systolic volume (Volume;s); LV end-diastolic volume (Volume;d); thickness of the LV anterior wall at end-systole and end-diastole (LVAW;s and LVAW;d) and thickness of the LV posterior wall at end-systole and end-diastole (LVPW;s and LVPW;d); stroke volume (SV); and cardiac output (CO) [17].

### 4.5. Histology

Formalin-fixed heart samples were processed, embedded in paraffin, and sectioned at a 5 μm thickness from the mid-ventricular region. Picrosirius red staining was used to evaluate cardiac fibrosis. A total of 15–20 fields were randomly selected per mouse using a Nikon Labophot microscope at 10× magnification with an AmScope camera (Nikon Instruments, Melville, NY, USA). Fibrosis was quantified as a percentage area using the ratio of Picrosirius red-stained area to the total myocardial area using ImageJ (v1.54k) software.

### 4.6. Statistical Analysis

Data are presented as mean ± SEM. Data were tested for normality by using the Shapiro–Wilk test. Comparisons between multiple groups at a single time point were performed using a One-Way ANOVA with Tukey’s post hoc test. Comparisons between multiple groups over time were performed using a Two-Way ANOVA. A *p* value < 0.05 was considered statistically significant.

## 5. Conclusions

Here, we showed that mutation of p53 acetylation is protective against Ang-II infusion-induced cardiac fibrosis and dysfunction, suggesting a novel role for p53 acetylation in the pathophysiology of hypertension. Acetylation of p53 may, therefore, be a novel therapeutic target to treat cardiac complications in patients with hypertension, although extensive additional investigation is required to determine the precise pathways that may be of therapeutic value.

## Figures and Tables

**Table 1:** Echocardiographic measurements of control (Con) and p53aceKO (pKO) mice receiving either sham or Ang-II infusion at week 4. Data are presented as mean ± SEM. N = 6 mice per group; ns = not significant, * *p* < 0.05, ** *p* < 0.01, **** *p* < 0.0001 using Two-Way ANOVA. LVAW;s, left ventricular anterior wall at end-systole; LVAW;d left ventricular anterior wall at end-diastole; LVPW;s, left ventricular posterior wall at end-systole; LVPW;d, left ventricular posterior wall at end-diastole.

	Con + Sham	Con + Ang-II	pKO + Sham	pKO + Ang-II	Control + Sham v p53aceKO + Sham	Control + Sham v Control + Ang-II	p53aceKO + Sham v p53aceKO + Ang-II
**CO (mL/min)**	15.5 ± 1.4	13.5 ± 2.3	20.9 ± 0.8	23.0 ± 2.0	*	ns	ns
**SV (uL)**	33.9 ± 3.0	29.5 ± 4.9	46.4 ± 1.9	49.9 ± 4.0	*	ns	ns
**LVAW;s (mm)**	1.32 ± 0.04	1.88 ± 0.05	1.71 ± 0.14	1.55 ± 0.10	ns	****	ns
**LVAW;d (mm)**	1.04 ± 0.06	1.33 ± 0.07	1.12 ± 0.08	1.02 ± 0.05	ns	*	ns
**LVPW;s (mm)**	1.17 ± 0.07	1.80 ± 0.10	1.36 ± 0.10	1.53 ± 0.17	ns	**	ns
**LVPW;d (mm)**	0.84 ± 0.04	1.21 ± 0.07	0.93 ± 0.07	1.05 ± 0.10	ns	**	ns

## Data Availability

Data are contained within the article.
